# Rationale and design of the Medical Research Council's Precision Medicine with Zibotentan in Microvascular Angina (PRIZE) trial

**DOI:** 10.1016/j.ahj.2020.07.007

**Published:** 2020-11

**Authors:** Andrew J Morrow, Thomas J Ford, Kenneth Mangion, Tushar Kotecha, Roby Rakhit, Gavin Galasko, Stephen Hoole, Anthony Davenport, Rajesh Kharbanda, Vanessa M Ferreira, Mayooran Shanmuganathan, Amedeo Chiribiri, Divaka Perera, Haseeb Rahman, Jayanth R. Arnold, John P. Greenwood, Michael Fisher, Dirk Husmeier, Nicholas A Hill, Xiaoyu Luo, Nicola Williams, Laura Miller, Jill Dempster, Peter W Macfarlane, Paul Welsh, Naveed Sattar, Andrew Whittaker, Alex Mc Connachie, Sandosh Padmanabhan, Colin Berry

**Affiliations:** a British Heart Foundation Glasgow Cardiovascular Research Centre, Institute of Cardiovascular and Medical Sciences, University of Glasgow, Glasgow, United Kingdom; b University of New South Wales, Sydney, Australia; c Royal Free Hospital, Royal Free London NHS Foundation Trust London, United Kingdom; d Lancashire Cardiac Centre, Blackpool Teaching Hospitals NHS Foundation Trust, Blackpool, United Kingdom; e Department of Interventional Cardiology, Royal Papworth Hospital, Cambridge, United Kingdom; f Experimental Medicine and Immunotherapeutics, University of Cambridge, Cambridge, United Kingdom; g Division of Cardiovascular Medicine, Radcliffe Department of Medicine, British Heart Foundation Centre of Research Excellence, NIHR Oxford Biomedical Research Centre, University of Oxford, John Radcliffe Hospital, Oxford, United Kingdom; h Department of Cardiology, Oxford University Hospitals NHS Foundation Trust, John Radcliffe Hospital, Oxford, United Kingdom; i Division of Imaging Sciences, Guy's and St Thomas' Hospital NHS Foundation Trust, London, United Kingdom; j School of Cardiovascular Medicine and Sciences, King's College London, London, United Kingdom; k Department of Cardiovascular Sciences, Glenfield Hospital, Leicester, United Kingdom; l Leeds University and Leeds Teaching Hospitals NHS Trust, Leeds, United Kingdom; m Liverpool University and Liverpool University Hospitals NHS Foundation Trust, Liverpool, United Kingdom; n School of Mathematics & Statistics, University of Glasgow, Glasgow, United Kingdom; o Department of Clinical Genetics, Queen Elizabeth University Hospital, Glasgow, United Kingdom; p Emerging Innovations Unit, Discovery Sciences, R&D, AstraZeneca, Cambridge, United Kingdom; q Robertson Centre for Biostatistics, Institute of Health and Wellbeing, University of Glasgow, Glasgow, United Kingdom

## Abstract

Microvascular angina is caused by cardiac small vessel disease, and dysregulation of the endothelin system is implicated. The minor G allele of the non-coding single nucleotide polymorphism (SNP) rs9349379 enhances expression of the endothelin 1 gene in human vascular cells, increasing circulating concentrations of ET-1. The prevalence of this allele is higher in patients with ischemic heart disease. Zibotentan is a potent, selective inhibitor of the ET_A_ receptor. We have identified zibotentan as a potential disease-modifying therapy for patients with microvascular angina.

**Methods:**

We will assess the efficacy and safety of adjunctive treatment with oral zibotentan (10 mg daily) in patients with microvascular angina and assess whether rs9349379 (minor G allele; population prevalence ~36%) acts as a theragnostic biomarker of the response to treatment with zibotentan.

The PRIZE trial is a prospective, randomized, double-blind, placebo-controlled, sequential cross-over trial. The study population will be enriched to ensure a G-allele frequency of 50% for the rs9349379 SNP. The participants will receive a single-blind placebo run-in followed by treatment with either 10 mg of zibotentan daily for 12 weeks then placebo for 12 weeks, or vice versa, in random order. The primary outcome is treadmill exercise duration using the Bruce protocol. The primary analysis will assess the within-subject difference in exercise duration following treatment with zibotentan versus placebo.

**Conclusion:**

PRIZE invokes precision medicine in microvascular angina. Should our hypotheses be confirmed, this developmental trial will inform the rationale and design for undertaking a larger multicenter trial.

## Background

### Microvascular angina

The term angina pectoris is derived from Latin, meaning “strangling of the chest”.^1^ Angina is a consequence of myocardial ischemia caused by an imbalance between myocardial blood supply and oxygen demand.^2^ Classically the management of patients with angina pectoris focuses on detection and treatment of flow-limiting epicardial coronary artery disease (CAD).^3^ However, fewer than half of patients with suspected angina have evidence of obstructive CAD on coronary angiography.^4^^,^^5^ Many patients with “normal” coronary angiography have signs, symptoms and investigations in-keeping with myocardial ischemia –this is termed ischemia with non-obstructive coronary artery disease (INOCA).^6^

INOCA is a heterogeneous group of conditions, including disorders of coronary anatomy, endothelial function and vasoreactivity.^7^ A growing body of evidence supports the differentiation of this group into distinct disease endotypes—the most common being microvascular angina and epicardial coronary vasospasm—that are amenable to stratified medicine.^3^^,^^8^

Microvascular angina is caused by coronary microvascular dysfunction. The Coronary Vasomotion Disorders International Study (COVADIS) Group define microvascular angina as symptoms of myocardial ischemia with proven coronary microvascular dysfunction (e.g. index of microcirculatory resistance (IMR) ≥25, coronary flow reserve (CFR) <2.0 or abnormal microvascular constriction during acetylcholine infusion), with unobstructed epicardial coronary arteries. (Table I).^9^

**Table I:** Coronary Vasomotion Disorders International Study (COVADIS) clinical criteria for the diagnosis of microvascular angina

COVADIS criteria for microvascular angina	
1. Symptoms of myocardial ischemia	• Effort and/or rest angina • Angina equivalents (i.e. shortness of breath)
2. Absence of obstructive CAD (>50% diameter reduction and/or FFR <0.80) by either:	• CT coronary angiography (CTCA) • Invasive coronary angiography
3. Objective evidence of myocardial ischemia	• Ischemic ECG changes during an episode of chest pain • Stress-induced chest pain and/or ischemic ECG changes in the presence of transient/reversible abnormal myocardial perfusion and/or wall motion abnormality
4. Evidence of impaired coronary microvascular function	• Impaired coronary flow reserve (cut-off values depending on methodology use between ≤2.0 and ≤ 2.5) • Coronary microvascular spasm, defined as reproduction of symptoms, ischemic ECG shifts but no epicardial spasm during acetylcholine testing. • Abnormal coronary microvascular resistance indices (e.g. IMR >25, HMR ≥2.5 mm Hg·cm-1·s)^69^ • Coronary slow flow phenomenon

The pathogenesis of microvascular angina is multi-factorial and incompletely understood. It may involve structural and/or functional abnormalities of the microvasculature. Standard invasive coronary angiography lacks the spatial resolution to image the microcirculation directly.^10^ However, microvascular remodeling, with decreased luminal size and rarefaction (loss of capillaries) is implicated.^11^ Functional microvascular angina involves enhanced vasoconstriction and/or impaired coronary vasodilatation in response to physiological (e.g. exercise) or pharmacological e.g. acetylcholine, adenosine, stimuli of the coronary microvasculature, with endothelium-dependent and independent mechanisms being demonstrated.^12^

### Zibotentan

Endothelin-1 (ET-1) is a small peptide, produced primarily in the endothelium that is a potent constrictor of human blood vessels, and importantly is the most potent vasocontrict0r of the coronary arteries.13., 14., 15., 16. ET-1 is mediated by two receptors: ET_A_ and ET_B_. ET_A_ activation by ET-1 mediates coronary vasoconstriction.17., 18., 19.

Microvascular angina is associated with elevated circulating concentrations of ET-1 and prolonged exposure to ‘excess’ endothelin has harmful effects on small vessel function.^20^^,^^21^ Additionally, ET-1 mediates enhanced vasoconstriction in the peripheral arterioles of patients with microvascular angina compared to control subjects.^22^

Two trials of ET-1 receptor antagonists in patients with microvascular angina reported favorable, preliminary results (Reriani NCT00271492; Johnson NCT00738049). However, these trials were limited in size and unfortunately both atrasentan (hormone-refractory prostate cancer) and darusentan (resistant hypertension) were subsequently discontinued following neutral results from phase III trials relating to their primary indications, thus stopping any further investigation of their potential application in microvascular angina.^23^^,^^24^

There are theoretical benefits to selective ET_A_ receptor antagonism in cardiovascular disease, given that non-selective ET_A_ and ET_B_ receptor antagonists in heart failure cause deleterious fluid retention and adverse outcomes. Supportive evidence of ET_A_ selective antagonism can be drawn from studies in the population with heart failure and preserved ejection fraction (HFpEF). The natural history of these conditions is linked with microvascular angina reflecting chronic myocardial ischemia that may lead to HFpEF in the longer term.^25^ A double-blind, placebo-controlled, randomized controlled trial of sitaxentan in patients with HFpEF disclosed improvements in exercise tolerance conferred by this ET_A_ receptor antagonist.^26^

Zibotentan is a potent inhibitor of the ET_A_ receptor with no “off-target” binding to the ET_B_ receptor.^27^ We have identified zibotentan as a potential disease-modifying therapy for patients suffering from microvascular angina; however, it has not been used before in this patient group and is currently unlicensed. Zibotentan was initially developed as a possible treatment for cancer, but it did not improve survival in this group of patients.^28^ With the support of the AstraZeneca Open Innovation program, we have developed a clinical pathway for repurposing zibotentan as a novel treatment for patients with microvascular angina. The safety, pharmacokinetics (PK), pharmacodynamics (PD) and efficacy of orally administered zibotentan have been examined in 20 Phase I studies, 4 Phase II studies and 3 Phase III studies.^29^

The dose being used in this study is 10 mg once daily (having been previously studied at doses up to 240 mg in oncology studies) to achieve adequate target coverage over a 24-hour period, whilst minimizing side effects relating to ‘target engagement’ which include headache, nasal congestion/rhinitis, nausea and/or vomiting, and peripheral edema.^27^^,^^30^ Importantly, zibotentan has been tested ex vivo at these concentrations and has been shown to fully reverse an established ET-1 vasoconstriction, indicative of efficacy in conditions associated with vasospasm.^31^ Clearance of zibotentan is reduced in patients with hepatic or renal impairment.^30^ Zibotentan carries a risk of fetal toxicity and a theoretical risk of transfer via semen or breast milk.^29^ Clinical risk in PRIZE will be mitigated by the exclusion of patients with a medical diagnosis that is associated with toxicity and specific guidance regarding women of child-bearing potential (WoCBP) and their partners. (Table II).

**Table II:** Exclusion criteria.

Exclusion criteria
1. Exercise tolerance >540 seconds in men and> 430 seconds in women during Full Bruce protocol or, lack of anginal symptoms and/or ST-segment depression (≥0.1 mV) limiting exercise.
2. Non-cardiovascular exercise-limiting problem e.g. morbid (or severe) obesity (BMI ≥40.0 kg/m^2^)
3. Genotype not available
4. Women who are pregnant, breast-feeding or of child-bearing potential (WoCBP) without a negative pregnancy test
5. Men who are sexually active with a WoCBP who are unwilling to highly effective methods of contraception for the duration of study treatment and for 14 weeks after the last dose of study drug.
6. Heart failure (New York Heart Association Grade ≥ II, i.e. mild symptoms and slight limitation during ordinary activity)
7. Recent (<3 months) myocardial infarction
8. A history of epilepsy, other CNS adverse events, neurologic symptoms or signs consistent with spinal cord compression or CNS metastases.
9. Moderate or more severe renal impairment (GFR <45 mL/min)
10. Liver disease with a Child-Pugh score of A (5–6 points) or higher
11. Participation in another intervention study involving a drug within the past 90 days

### Precision medicine in microvascular angina

The chronic elevation of circulating ET-1 in microvascular angina may be influenced by genetic factors. A gene variant of interest is rs9349379—this is a common non-coding single-nucleotide polymorphism (SNP) in the third intron of the protein-coding PHACTR1 gene on chromosome 6.^32^ This SNP regulates expression of the Endothelin 1 (EDN1) gene in human vascular cells which encodes the ET-1 protein. The minor G allele of this SNP (population prevalence ~36%) is associated with significantly increased circulating concentrations of ET-1.^33^

Emerging evidence from our laboratory (BHF CorMicA ClinicalTrials.gov: NCT03193294) indicates the prevalence of this SNP may be more common in patients with microvascular angina than is observed in age- and sex-matched controls. Patients with the rs9349379 G allele had higher serum ET-1 and over double the odds of coronary microvascular dysfunction. Additionally, subjects were more likely to have impaired myocardial perfusion and reduced exercise tolerance.^31^

### Hypothesis

**Figure 1:**
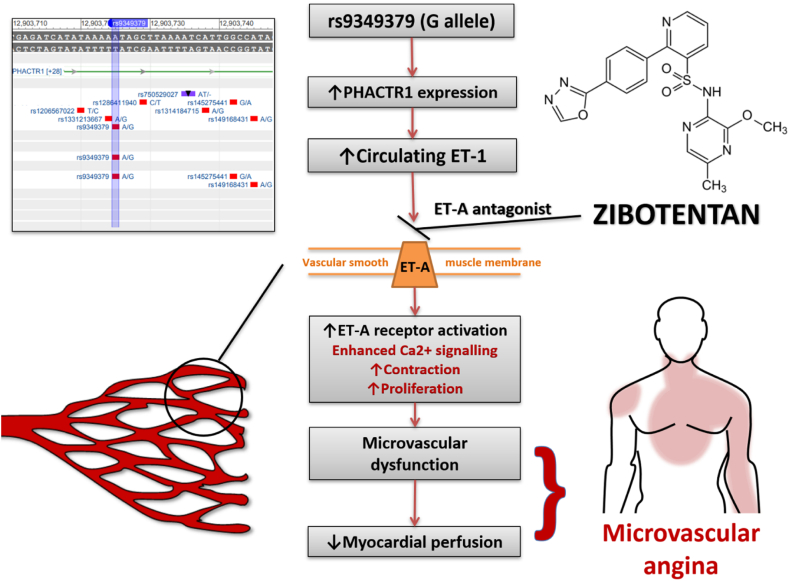
Summary of the rationale for treatment with an ET_A_ antagonist in microvascular angina and for rs9349379 as theragnostic biomarker.

Our hypothesis is that the ET_A_ antagonist, zibotentan, will be an effective treatment for patients with microvascular angina. We further hypothesize that the SNP regulator of EDN1 gene expression, rs9349379 (minor G allele), will act as a novel genomic theragnostic biomarker that associates with treatment response in this patient group. (Figure 1).

### Aims

1. To assess the efficacy of adjunctive treatment with zibotentan in patients with microvascular angina.
2. To explore the mechanisms of any beneficial effects of zibotentan in microvascular angina.
3. To assess genetic and clinical biomarkers for baseline patient characteristics and treatment response.
4. To determine rates and severity of adverse events associated with zibotentan and evaluate its safety in a non-oncology population
5. To explore the feasibility of the personalized medicine strategy in an ischemic heart disease population.

### Study design

**Figure 2:**
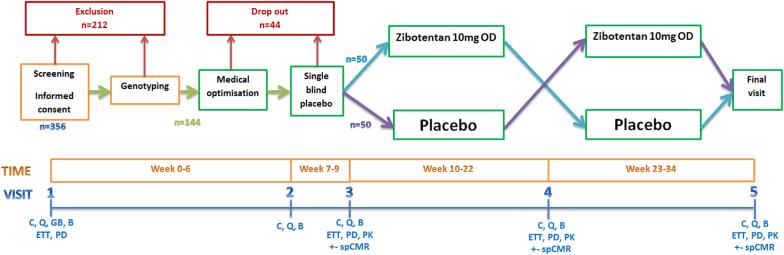
Schematic study design: flow diagram. Abbreviations: Blood tests (B), Clinical review (C), Exercise tolerance test (ETT; Bruce protocol), Genomic blood test (GB), Questionnaires (Q), Stress perfusion cardiac magnetic resonance imaging (spCMR; optional sub-study), Pharmacokinetic and pharmacodynamic sampling (PK/PD).

The PRIZE trial has a prospective, randomized, double-blind, placebo-controlled, sequential cross-over design. The study population will be enriched to ensure the G-allele frequency of the rs9349379 SNP is at least 50%. Participants will receive a single-blind run-in with placebo followed by double-blind treatment with either 10 mg of zibotentan daily for 12 weeks then placebo for 12 weeks, or vice versa, in random order. The trial is designed to assess the superiority of the addition of oral zibotentan to guideline-indicated therapy as compared with placebo and guideline-indicated treatment for patients with microvascular angina. (Figure 2).

### Setting

The trial will involve multiple centers in the UK (Queen Elizabeth University Hospital (Glasgow), Royal Free Hospital (London), Blackpool Teaching Hospitals, Royal Papworth Hospital (Cambridge), Department of Cardiology in the Oxford University Hospitals NHS Foundation Trust and Division of Cardiovascular Medicine at the University of Oxford, John Radcliffe Hospital (Oxford), Guy's & St. Thomas' Hospital (London), University Hospitals of Leicester, Liverpool University Hospitals Foundation NHS Trust, (Liverpool) and the Leeds Teaching Hospitals NHS Trust. Additional sites may be included in order to ensure recruitment milestones are achieved.

### Patient identification

Patients will be identified from clinical databases, clinics and clinical procedure lists. The clinical pathways include but are not limited to: (1) out-patient clinics including patients with a diagnosis of microvascular angina; (2) diagnostic stress tests, e.g. stress perfusion magnetic resonance imaging (MRI), stress echocardiography, stress nuclear imaging with PET or SPECT or an exercise ECG leading to a diagnosis of microvascular angina; (3) invasive or CT coronary angiography.

### Eligibility criteria

The inclusion criteria are: (1) age >18 years old; (2) microvascular angina; (3) able to comply with study procedures; (4) able to provide written informed consent.

In PRIZE, we define microvascular angina as per the COVADIS guideline (Table I). The criteria are:

1. Symptoms of myocardial ischemia.
2. Absence of obstructive CAD (>50% diameter reduction or FFR <0.80) by either CT or invasive coronary angiography.
3. Objective evidence of myocardial ischemia.
4. Evidence of impaired coronary microvascular function.

In order to participate in this trial, the patient should have a diagnosis of microvascular angina (probable or definite). Probable microvascular angina is defined as having 3 of the 4 COVADIS criteria. Definite microvascular angina requires all 4 COVADIS criteria. Participants in this trial should also fulfill criteria 1 & 2. This diagnostic classification is endorsed by the European Society of Cardiology practice guidelines (August 2019).^3^

The exclusion criteria are listed in Table II

### Recruitment

Three hundred fifty-six patients will be enrolled following written informed consent. Those eligible on clinical grounds will undergo genotype blood testing. All those with the GG genotype will continue to the run-in phase, whereas only a proportion of those with the AA and AG genotypes will proceed. This will boost the relative frequency of the G genotypes in the study population, with the objective of achieving 50% G allele frequency. If a consented patient is found to be ineligible for the run-in/treatment phase of the randomized trial, they will remain included in the study population, including consent for long term follow up using linkage of electronic government and patient records (EPR).

Following genotyping and enrichment of the study population with the minor G allele for the rs9349379 SNP we anticipate enrolling at least 144 patients into the run-in phase of the study. Allowing for up to 30% drop-out during run-in, at least 100 participants will proceed to randomization.

### Genotyping

Genomic DNA will be extracted from a peripheral blood sample (EDTA containing tube taken during Visit 1). A Sanger sequencing approach, using the forward primer “F_GTGCAATTCTCCAAGGCTCC” and the reverse primer “R_TTTAAAACTCAGCTCGTGGAAAA”, will be used to sequence part of intron 3 of the PHACTR1 gene to determine the genotype of the rs9349379 SNP.

### Medical optimization

Since microvascular angina is a chronic condition, most patients will already be established on maintenance drug therapy. In some cases, cardiovascular risk factors, including blood pressure and lipids, may not be optimally managed. To accurately assess the efficacy of zibotentan, we will first ensure that patients recruited to the study are being managed with guideline-directed medical therapy as per contemporary practice guidelines and local standards of care. This process includes informed consent (Visit 1) through to treatment run-in (Visit 2). The medical optimization period is intended to identify patients with unstable symptoms or uncontrolled cardiovascular risk factors. In affected cases, optimization of pharmacological and non-pharmacological treatments and will be carried out by clinical staff including clinical research nurses, study physicians and pharmacists, as clinically appropriate.

The clinical review and optimization process will follow a standard operating procedure (SOP), in line with contemporary clinical practice guidelines, notably those issued by the European Society of Cardiology.^3^ For logistical reasons, this optimization phase is limited to 6 weeks. If angina drug therapy is changed, then a period of 4 weeks will follow before proceeding into the treatment run-in phase. Following optimization, angina therapy will remain the same from entry into the treatment run-in period (Visit 2) and thereafter.

## Outcomes

### Primary outcome

The primary outcome for our study is the treadmill exercise time (seconds) using the full Bruce exercise tolerance test protocol.^34^ Exercise testing using the Bruce protocol is a standard of care in clinical cardiology and evidence-based for assessing functional capacity, susceptibility to effort-related anginal symptoms and ischemia in patients with stable angina.^35^ Treadmill exercise time (s) is a reproducible outcome measure35., 36., 37., 38., 39., 40., 41., 42. with an approximately 10% test-retest variability.^36^^,^^42^ In a study of repeated exercise testing in older women the intra-class correlation coefficient of exercise duration was 0.88.^38^ In a clinical trial involving 33 patients with microvascular angina, there was 100% compliance with serial exercise tests (n = 4 per subject). Patient and Public Involvement (PPI) in the design of this study provided encouraging feedback to the effect that exercise tests are generally well tolerated. Treadmill exercise testing is also accepted by regulators, such as the Federal Drug Administration, for assessing the efficacy of angina medications.

### Secondary outcomes

The pre-specified secondary outcomes are described in Table III.

**Table III:** Secondary outcomes.

Secondary outcomes	
Exercise test	• Time (s) to 1 mV ST depression
	• Maximum ST-segment deviation (mV)
	• Time (s) to 75% of max age-related heart rate during exercise
	• Metabolic equivalent (METs)
	• The DUKE Score^70^
Health Status Questionnaires	• Seattle Angina Questionnaire (SAQ)^43^
	• Illness perception • (Brief IPQ)^48^
	• Anxiety/depression • (PHQ4)^50^
	• Treatment satisfaction • (TSQM)^49^
	• EQ-5D-5L^47^
Safety	• Frequency and severity of severe adverse events (SAEs)
Feasibility	• Participant withdrawal rate
Efficacy	• Pharmacokinetics • Pharmacodynamics
Exploratory endpoints/outcomes	
Patient-Reported Outcome Measures (PROMS)	• Angina diary

The primary and secondary outcomes, and the MRI sub-study, are intended to provide surrogate outcome information that is mechanistically linked to the pathogenesis of microvascular angina. Accordingly, the results of our precision medicine trial should be informative about the potential role for zibotentan as a disease-modifying therapy for microvascular angina.

### Questionnaires

The Seattle Angina Questionnaire (SAQ) is a validated, disease-specific questionnaire that quantifies limitations caused by angina, the frequency of angina, treatment satisfaction, and subjective perception of quality of life.^43^ Each component score is converted and collated to give a total score out of 100, where a higher score indicates better function. SAQ scores are independently associated with mortality, hospitalization, and resource use.44., 45., 46. The SAQ is a sensitive instrument in patients with microvascular angina.^8^

Self-reported health status will be assessed using the generic EuroQOL EQ-5D-5L questionnaire and the Brief Illness Perception Questionnaire (Brief-IPQ).^47^^,^^48^ The Treatment Satisfaction Questionnaire (TSQM-9) will provide information regarding medication side effects, effectiveness, convenience and overall satisfaction.^49^We will also utilize the PHQ–4 questionnaire that is a validated tool for detecting both anxiety and depressive disorders.^50^ Participants will be invited to complete these questionnaires at each visit.

### Pharmacokinetics

Blood samples will be obtained during visits 3, 4, and 5 (Figure 2) to measure steady-state plasma concentrations of zibotentan reflecting chronic treatment with the last dose being the preceding day.

Adherence with trial medication will be assessed by participant-reported adherence with therapy (number of days with a missed dose during the preceding phase; duration of the phase (days); number of days with a missed dose in the past 7 days), and time of the last dose prior to the visit).

### Pharmacodynamics

In order to research the mechanisms of any potential benefit of oral zibotentan on the described endpoints, we will compare the within-subject differences in the circulating concentrations of soluble intercellular adhesion molecule-1 (sICAM-1), high sensitivity (hs) interleukin-6 (hsIL-6), ET-1, hs-troponin I, NT-proBNP, cystatin C, lipid profile, glucose, HbA1c, and hsCRP.

The associations between the within-subject differences in circulating concentrations of these mechanistic biomarkers and the primary and secondary outcomes for efficacy and safety will be assessed.

### Cardiovascular magnetic resonance imaging (MRI) sub-study

Myocardial perfusion is generally impaired in patients with microvascular angina. The rational for undertaking the MRI sub-study is to determine whether, compared with placebo, treatment with zibotentan improves myocardial blood flow. Adenosine stress perfusion MRI is scheduled for 3 occasions in 60 participants. A detailed description of the sub-study is beyond the scope of this article and will be described separately in a future article.

### Angina diary

The diagnostic performance of a custom-developed questionnaire for symptoms, quality of life and response to treatment will be assessed as an exploratory outcome. Participants will be invited to complete this diary each time symptoms occur during the trial.

### Feasibility

This study has been peer reviewed by panel members of the Medical Research Council (MRC) Biomedical Catalyst Developmental Pathways Scheme and the MRC Stratified Medicine Board. Feedback has also been provided by the British Society of Cardiovascular Magnetic Resonance Research Group, the British Cardiovascular Society and the Cardiovascular Care Partnership (UK).

## Statistical considerations

### Sample size calculation

The primary outcome is the treadmill exercise time (seconds). A 30-second difference in exercise duration is considered clinically significant.51., 52., 53. The standard deviation of the difference between two exercise test measurements is assumed to be 85 seconds.^51^ To achieve 80% power to detect a mean difference of 30s between treatments in a 2 × 2 crossover design and a level of significance of 0.05 (alpha error) requires complete data in 65 subjects. At least 100 subjects will be randomized to allow for data quality issues and loss to follow-up. Considering the clinical optimization (visit 1–2) and single-blind placebo run-in (visits 2 and 3), we anticipate a drop-out of up to 30% may occur (n = 42), meaning we will aim to have 144 begin the run-in phase of the trial to randomize 100.

Pre-specified subgroup analyses are intended for sex, a history of vasospastic angina, and the duration of symptoms.

### rs9349379 SNP G-allele enrichment

The study population will be enriched to achieve approximately 50% prevalence of the G allele of the rs9349379 SNP, by selectively including patients with the AA and AG genotypes.

The sampling rates of AA and AG patients will be set at the start of the trial, based on expected allele frequencies, but will be monitored during the trial, and may be modified, based on the genotype distribution achieved. This process will be monitored by the Trial Steering Committee. The enrichment process will be balanced against the rate of recruitment into the study, and if recruitment falls behind timelines, the sampling rates may be modified to increase the numbers randomized, at the expense of having a lower than 50% G allele frequency.

### Run-in phase

Participants will enter a three-week run-in phase from Visit 2 to Visit 3. Participants will receive a once daily single blind placebo medication. The purpose of this run-in period is to give the participants experience of taking a study medication. Since assessments of adherence with study therapy and safety are objectives of the trial, we purposefully decided against a run-in phase with zibotentan, since participants who might be intolerant of the active treatment would then not be included in the trial.

### Randomization

Randomization will occur during Visit 3, following successful completion of a single-blind placebo run-in. Eligible and consenting patients will be randomized with equal probability to the two groups reflecting the sequential order of zibotentan or placebo in Phase 1 and Phase 2, respectively: Group 1 = zibotentan in Phase 1 then placebo in Phase 2; Group 2 = placebo in Phase 1 then zibotentan in Phase 2. The randomization will be minimized with respect to a concomitant history of vasospastic angina, study site, genotype, and sex.

### Blinding

The study is double-blind. Specifically, the trial participants, carers, clinical staff, and researchers will be blinded to the intervention. Outcome assessments (endpoint adjudication) will be undertaken in blinded fashion.

Breaking of the study blinding in an emergency will only be performed where knowledge of the treatment is absolutely necessary for further medical management of the patient. Any emergency unblinding will occur via a telephone Interactive Voice Response System (IVRS). Unblinding the treatment allocation may be required when reporting suspected unexpected serious adverse reactions (SUSARs) to the regulatory authorities. This will be performed by the sponsor pharmacovigilance office.

## Trial management and governance

### Trial management

The trial will be conducted in line with the current *Guidelines for Good Clinical Practice in Clinical Trials* and CONSORT guidelines.^54^^,^^55^

The Trial Management Group (TMG) includes those individuals responsible for the day-to-day management of the study including the chief investigator, project manager and representatives from the co-sponsors. The role of this group will be to facilitate the progress of the study, ensure that the protocol is adhered to and take appropriate action to safeguard participants and the quality of the study itself.

A Trial Steering Committee (TSC) compromising of an independent chairperson, two independent cardiologists, a representative from the MRC and a patient representative has been formed to provide overall supervision of the trial and ensure that it is being conducted in accordance with the principles of GCP and the relevant regulations. Decisions about continuation or termination of the trial or substantial amendments to the protocol will be the responsibility of the TSC who will advise the co-sponsors.

An Independent Data Monitoring Committee (IDMC) including two independent medical experts and an independent biostatistician will receive unblinded reports on study safety data and on study progress/outcomes. The IDMC may recommend to the TSC and co-sponsors that the study should stop prematurely because of concerns about patient safety.

Since the trial is a crossover trial, and not designed to assess between-group differences in clinical endpoints, a Clinical Event Committee is not required. The authors are solely responsible for the design and conduct of this study, all study analyses, the drafting and editing of the paper and its final contents.

### Ethics

The PRIZE trial is approved by the UK National Research Ethics Service (Reference 19/NE/0110).

### Sources of funding

PRIZE is an investigator-initiated clinical trial that is funded by the Medical Research Council (MR/S018905/1). The funder is represented on the TSC, but the representative has no role in the trial design, conduct (non-voting TSC member), data analysis and interpretation, manuscript writing, or dissemination of the results.

AstraZeneca has provided the investigational medicinal product (IMP). AstraZeneca reviewed and approved the protocol. The company does not have any role in the conduct of the trial, analysis of the data, or publication of the results.

The MRI sub-study will involve imaging and analyses technologies provided by Siemens Healthcare.

The trial is co-sponsored by NHS Greater Glasgow & Clyde and the University of Glasgow. C.B. is supported by funding from the British Heart Foundation (RE/18/6134217).

### Registration

The ClinicalTrials.gov registration for this study is NCT04097314

### Statement of informed consent

Written informed consent is an eligibility criterion and is required before any study assessments are undertaken. Our informed consent form covers the genetic screening test for eligibility, the screening phase, the run-in-phase and the randomized study. Additionally, participants will be invited to provide optional consent for follow-up using electronic linkage of NHS and government records in the longer term. Ongoing consent will be confirmed during each study visit. Should consent be withdrawn then the participant will be withdrawn from the study without affecting her/his standard of care.

## Discussion

Precision medicine aims to individualize patient care bringing more effective therapy, while reducing both risks to patients and pressure on healthcare systems by avoiding unnecessary treatments and investigations.^56^ Recent position papers emphasize the vital role of genetic data, accurate diagnostic tests and novel pathophysiological insights in the development of precision cardiovascular medicine.^57^

In recent years, there have been significant advances in the understanding of the pathophysiology and diagnosis of microvascular angina. However, treatment has lagged behind. Currently, there are no disease-modifying therapies for microvascular angina.^3^^,^^58^^,^^59^ Instead, management remains mostly empirical with a “trial-and-error” approach being commonplace with an unsatisfactory trend towards polypharmacy and repeated clinic visits. Chronic, refractory symptoms are the norm. Historically, cognitive bias towards coronary heart disease (predominately focusing on epicardial coronary artery disease), combined with the lack of specific treatments for microvascular angina, has led to therapeutic nihilism amongst many clinicians.^60^

Phenome wide association studies (PheWAS) have provided insight on the polygenic nature of several common diseases and more than 100 genetic loci associated with CAD have already been identified.^61^^,^^62^ The rs9349379 gene variant provides an unique opportunity to enhance the therapeutic index of ET_A_ antagonist therapy and champions precision medicine.

To our knowledge, PRIZE is the first to apply pharmacogenomics to a randomized controlled trial in stable ischemic heart disease. As such, this experience will have transferable implications for clinical practice and trials in the future. We aim to advance precision medicine in microvascular angina through mechanistic outcomes. We will collect information on participants' characteristics at baseline including medical history, health status, medication, rs9349379 genotype, and exercise tolerance. The participants will be invited to give informed consent for life-long follow-up by linkage of electronic patient records. We have designed a bespoke angina diary that will provide quantitative data to correlate with the nature, frequency and severity of symptoms. Using multivariable analyses we will link baseline patient characteristics to treatment response with contemporary angina therapy.

Recent studies have challenged the long-held view that microvascular angina is a prognostically benign condition, reporting increased rates of major adverse cardiovascular events (MACE).^63^^,^^64^ New medicines are urgently required for these patients with an unmet clinical need. Should our hypotheses be confirmed, this developmental clinical trial will inform the rationale and design for undertaking a larger multicenter trial.

### Limitations

The protocol involves sequential treatment from Phase 1 to Phase 2 without a washout period. The duration of treatment (12 weeks) is longer than in pivotal trials of evidence-based anti-anginal drugs and should be sufficient to represent chronic maintenance therapy.^52^^,^65., 66., 67., 68. Realistically, the effects of treatment with zibotentan, notably attenuation of vasoconstriction, are unlikely to persist during chronic treatment with placebo for 12 weeks. There are also potential limitations with a washout period since it could increase the possibility of unblinding by giving the participants additional insights into the effects of the trial medication and the washout period would also prolong the trial.
